# Multidimensional competition of nematodes affects plastic traits in a beetle ecosystem

**DOI:** 10.3389/fcell.2022.985831

**Published:** 2022-08-24

**Authors:** Tess Renahan, Ralf J. Sommer

**Affiliations:** Max Planck Institute for Biology, Tübingen. Department for Integrative Evolutionary Biology, Tübingen, Germany

**Keywords:** resource competition, developmental plasticity, nematodes, Pristionchus pacificus, mouth-form polyphenism, dauer development

## Abstract

Resource competition has driven the evolution of novel polyphenisms in numerous organisms, enhancing fitness in constantly changing environmental conditions. In natural communities, the myriad interactions among diverse species are difficult to disentangle, but the multidimensional microscopic environment of a decaying insect teeming with bacteria and fighting nematodes provides pliable systems to investigate. Necromenic nematodes of the family Diplogastridae live on beetles worldwide, innocuously waiting for their hosts’ deaths to feast on the blooming bacteria. Often, more than one worm species either affiliates with the insect or joins the microbial meal; thus, competition over limited food ensues, and phenotypic plasticity provides perks for species capable of employing polyphenisms. The recently established system of cockchafer *Gymnogaster bupthalma* and its occasional co-infestation of *Pristionchus mayeri* and *Acrostichus spp*. has revealed that these worms will simultaneously utilize two polyphenisms to thrive in a competitive environment. While both genera maintain plastic capacities in mouth form (strictly bacterial-feeding and omnivorous predation) and developmental pathway (direct and arrested development, dauer), *P. mayeri* employs both when faced with competition from *Acrostichus*. Here, we took advantage of the malleable system and added a third competitor, model nematode *Pristionchus pacificus*. Intriguingly, with a third competitor, *P. mayeri* is quicker to exit dauer and devour available food, while *Acrostichus* hides in dauer, waiting for the two *Pristionchus* species to leave the immediate environment before resuming development. Thus, experimental manipulation of short-lived ecosystems can be used to study the roles of polyphenisms in organismal interactions and their potential significance for evolution.

## Introduction

“And striving to be man, the worm mounts through all spires of form.”

Ralph Waldo Emerson, 1867.

Poet Ralph Waldo Emerson in “May Day” lyrically acknowledged worms’ evolution of myriad forms, an early appreciation of phenotypic plasticity in a beloved metazoan (Emerson, 1867). Verily, phenotypic plasticity, the capacity of organisms to develop various phenotypes depending on environmental conditions, has been identified and detailed in numerous species across the domains. Favorite laboratory bacterium *Escherichia coli* displays morphological shape plasticity in response to physical stress (Matuła et al., 2019) and plasticity in cell division symmetry reflecting nutrient availability (Lele et al., 2011). The ability of an array of archaeal species thriving in wide habitat ranges, including extreme environments, is partially attributed to phenotypic plasticity (Banciu and Muntyan 2015; Choudoir et al., 2018). The phenomenon is rampant in Eukarya, with sundry studies detailing the intricacies at the genetic, molecular, and ecological levels and illuminating on how phenotypes evolve (Schlichting and Pigliucci 1998; West-Eberhard 2003; Pfennig 2021). Nematodes have been at the forefront of such investigations, particularly insect-associated *Pristionchus pacificus* (Hong and Sommer 2006; Sommer and McGaughran, 2013; Sommer 2015).

Ubiquitous *P. pacificus* maintains a necromenic relationship with hexapods globally (Herrmann et al., 2006, 2007), innocuously waiting for its host to die, ready to feast on the bacterial blooms decomposing the insect carcass. This association is not unique to *P. pacificus,* as many of the other 48 *Pristionchus* species affiliate with insects (Kanzaki et al., 2021), occasionally co-occurring not only in the same host species, but host individuals (Meyer et al., 2017; Renahan and Sommer, 2021). Upon multi-species infestation of the host, the worms are no longer harmless, at least with each other, as competition is incited. Edible resources on a decaying insect carcass are limited, provoking employment of combative strategies to ensure success. For example, entomopathogenic nematodes (EPNs) sustain an arsenal of schemes, including establishing their preferred symbiont while producing antibiotics to remove unwanted bacteria and fungi (Akhurst 1982; Kaya and Koppenhöfer 1996) and interspecific killing (Koppenhöfer et al., 1995; O’Callaghan et al., 2014; Zenner et al., 2014). Free-living worms wiggling their way to the bacterial buffet can interfere with EPNs’ dominance by both populating rapidly or surreptitiously dining amidst sparring, distracted EPNs (Duncan et al., 2003; Campos-Herrera et al., 2015a, Campos-Herrera et al., 2015b). In lieu of these strategies, nematodes armed with plasticity can tactically utilize specific phenotypes in order to protect their fitness.

**FIGURE 1:**
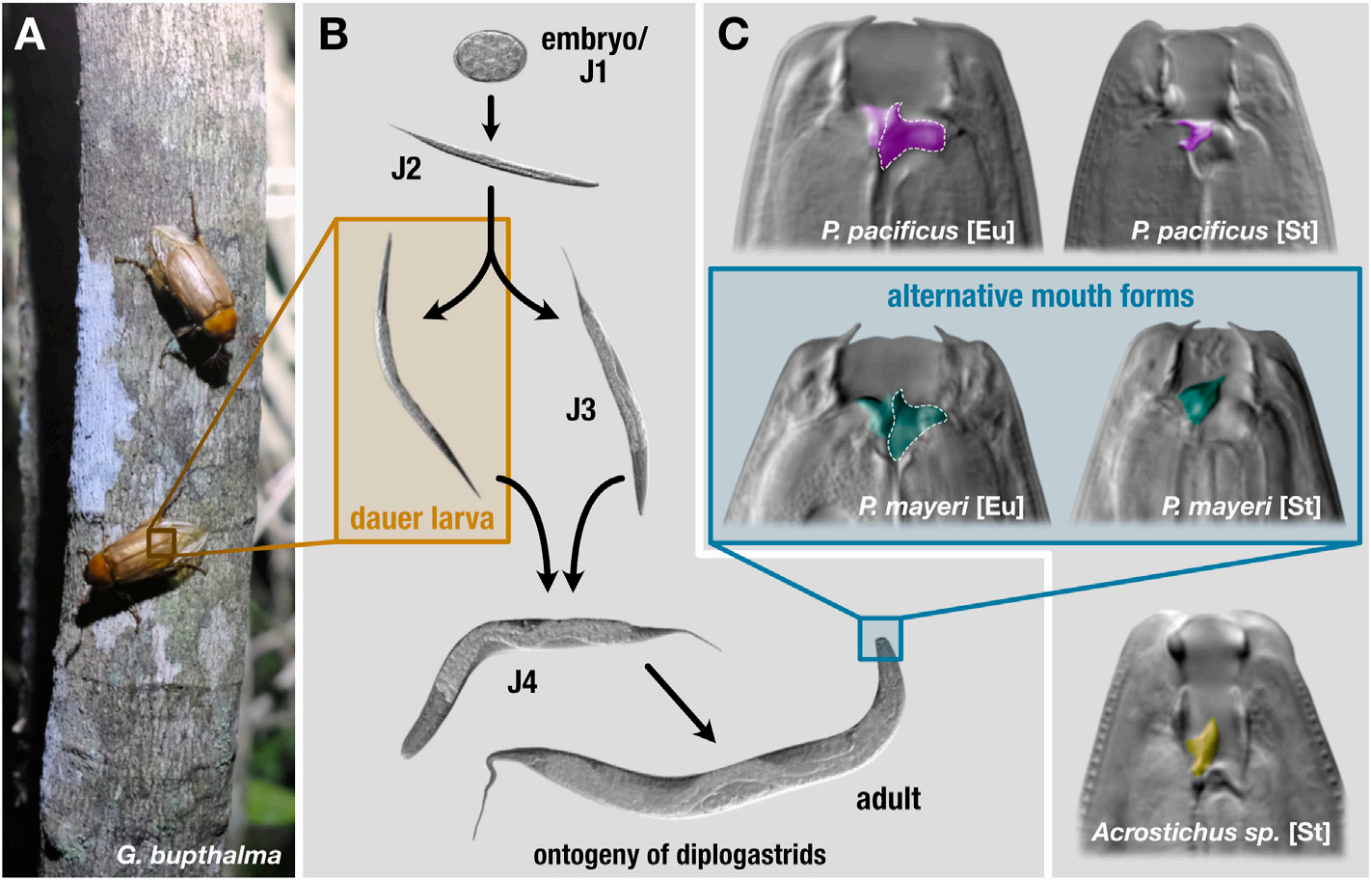
Lifecycle and mouth forms of three diplogastrids. On beetle hosts **(A)**, *P. pacificus* is found exclusively in the dauer stage. **(B)** Under favorable conditions, these nematodes develop from embryos to reproducing adults in roughly 4 days. In adverse environmental settings, young juveniles can enter an arrested developmental stage, dauer. Dauers are triggered to exit in improved conditions and resume development. **(C)** The mouth forms of the three diplogastrid species. *Pristionchus pacificus* and beetle-derived *P. mayeri* can develop either as bacterial-feeding stenostomatous (St) or predatory eurystomatous (Eu). Beetle-derived *Acrostichus* only developed as St. The teeth are colored by species. St worms only contain one tooth, the dorsal, while Eu have both the dorsal tooth and a subventral tooth, the latter of which is outlined in dotted white.

Phenotypic plasticity enables critical advantages in an arena scarce with sustenance and crowded with competitors. *Pristionchus pacificus* and other nematodes maintain an alternative developmental pathway that provides an escape from harsh environmental conditions. Under favorable settings, self-fertilizing hermaphroditic (with occasional males) *P. pacificus* develops from an embryo to an adult in roughly 4 days, but when faced with scant food, dense populations, or extreme temperatures, young juveniles can develop into dauer, an arrested, non-feeding developmental stage equipped with morphological and physiological alterations facilitating survival in stressful environments (Figure 1B) (Cassada and Russell, 1975; Riddle et al., 1981; Ogawa and Sommer, 2009). Upon improved conditions, dauer exit is triggered and worms continue developing into reproducing adults. Dauer is the only stage in which *P. pacificus* is found on live beetles (Herrmann et al., 2010; Meyer et al., 2017) and serves as the dispersal stage for both *P. pacificus* and *Caenorhabditis elegans* (Golden and Riddle, 1984). Thus, this plasticity affords both a spatial escape to better conditions and a temporal avoidance of adverse surroundings in anticipation of their amelioration. In addition to dauer, diverse diplogastrids display a mouth-form polyphenism in adults that affects dietary range: juveniles make an irreversible decision to become either stenostomatous (St), a single-toothed bacterivore, or eurystomatous (Eu) (Figure 1C), a two-teethed predator that can supplement its diet with fungi and other nematodes (Ragsdale et al., 2013; Susoy and Sommer, 2016). This broadened buffet enables Eu worms to eliminate their competition while simultaneously alleviating the need for the resource being fought over.

Whilst many molecular and genetic facets of dauer and mouth form have been extensively detailed in the lab, the significance of the two polyphenisms in a natural context has only recently begun to be explored. Worm-filled La Réunion Island in the Indian Ocean is a biodiversity hotspot with *Pristionchus*-infested beetles, allowing for extensive studies of its evolutionary ecology, population genetics, and role in ecosystem functioning (Morgan et al., 2014; McGaughran et al., 2016; Moreno et al., 2016). Initial investigations of the two polyphenisms in natural context focused on *P. pacificus*, utilizing its consistent infestation of abundant rhinoceros beetle *Oryctes borbonicus* (Meyer et al., 2017; Renahan et al., 2021), though this system lacks reliable co-occurrence of competing nematodes.

Recently, we established a novel nematode-beetle system, enabling examination of competing diplogastrids *Pristionchus mayeri* and *Acrostichus* as they strive to thrive post-death of their host, cockchafer *Gymnogaster bupthalma* (Figure 1A). Almost 30% of the endemic beetle is co-infested with the rival worms; we previously followed the dynamics of the two species after decapitation of the host (instigating nematode emergence) in a mock-natural setting in the laboratory. We found that *P. mayeri* responded to dense *Acrostichus* populations by developing the predatory morph in adults, in contrast to its preferential bacterivorous form, while *Acrostichus* did not exhibit the dimorphism and was exclusively stenostomatous, unable to predate, and fed only on bacteria and the remnants of nematode carcasses (Figure 1C) (Renahan and Sommer, 2021). In addition, *P. mayeri* displayed a dissimilar dispersal pattern to *P. pacificus* on *O. borbonicus*: the former maintained a constant population of both dauers and feeding stages on the decaying carcass, while *P. pacificus* displayed a biphasic “boom and bust” dynamic (Renahan et al., 2021). While *P. mayeri* is a self-fertilizing hermaphrodite with a small population of males similar to *P. pacificus*, the *Acrostichus* animals observed were gonochoristic (male/female), which may affect population dynamics.

**FIGURE 2:**
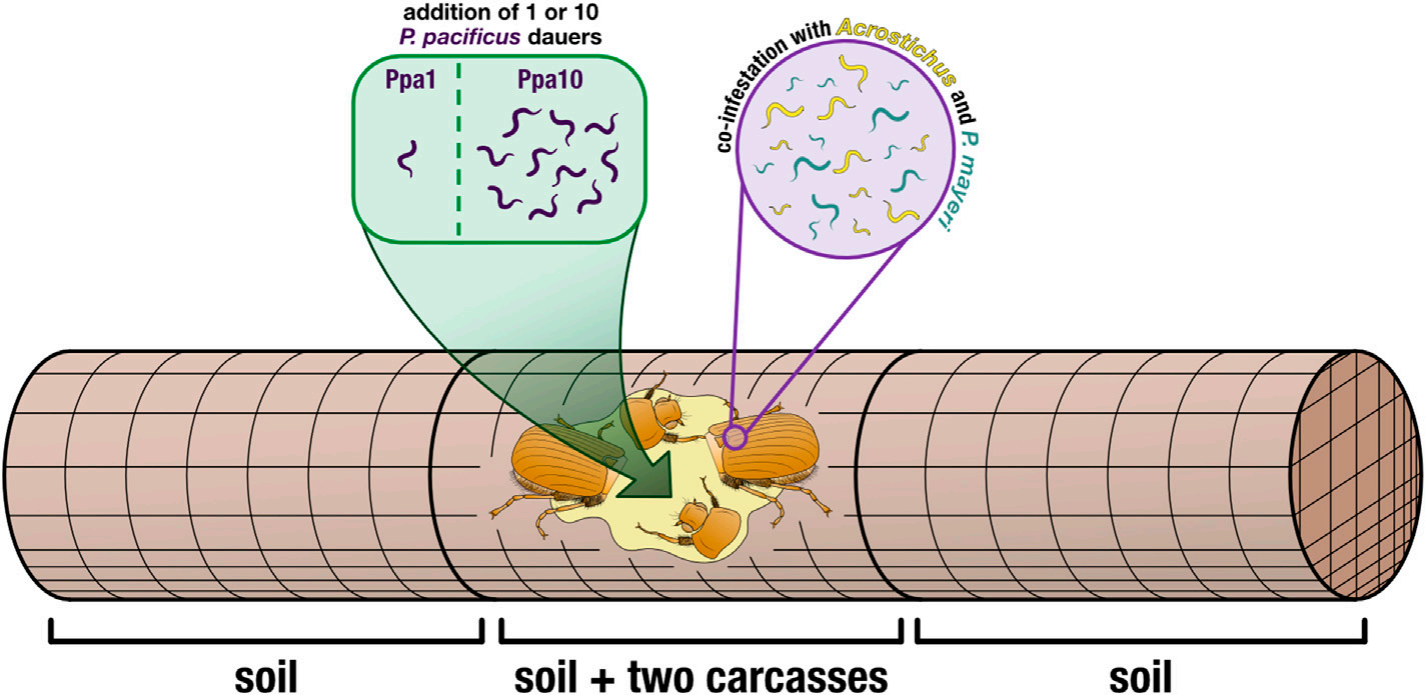
Cage set-up. Two decapitated beetles (to induce nematode emergence) were placed in the center of a wire-mesh cage (allowing for free movement of the worms). Cages either had one *P. pacificus* dauer added (Ppa1) or ten *P. pacificus* dauers added (Ppa10), directly to the carcasses. Wire-mesh dividers compartmentalized the carcasses in the middle, and the empty space was filled with soil. The rest of the cage (the two outer compartments) was also filled with soil, providing more space for the worms to disperse. Thus, worms were isolated from three locations: the carcasses, the soil in the carcass compartment, and the two soil compartments surrounding the carcass compartment.

Here, we sought to expand on these findings via experimental manipulation of the pliable system by adding a third competitor to *G. bupthalma* and observe how plastic traits are employed in an intricate beetle ecosystem. Consistent with established methods, we collected, decapitated, and placed adult *G. bupthalma* in soil-filled cages. To ensure each replicate was positive for both *P. mayeri* and *Acrostichus*, we added two dead beetles per cage (Figure 2). We then added either one or ten *P. pacificus* dauers to the freshly decapitated beetle pair using a fluorescently labeled strain to properly distinguish the two *Pristionchus* species. We tracked the dynamics of the three worm species over the course of 2 months. In both cases, *P. pacificus* abandoned its boom and bust strategy, and gradually populated while developing both St and Eu adults. Seemingly triggered by the presence of ten *P. pacificus* worms, *P. mayeri* both emerged from the carcass earlier, exited dauer quicker, and proliferated hastier, while *Acrostichus* ensconced in dauer until the other two species reduced their population sizes. Intriguingly, *P. mayeri* did not develop as predators in populations dense with *P. pacificus*, suggesting an alternative tactic to out-play its opponent, though as *Acrostichus* numbers rose, so did Eu *P. mayeri.* Thus, equipped with dauer and mouth form, worms can strategically employ these polyphenisms in the multidimensional competitive battleground of a decaying beetle carcass.

## Materials and methods

### Beetle collection, dauer induction of laboratory strain, and cage assembly

On La Réunion Island in January 2022, we caught adult male *G. bupthalma* flying as they sought mates at night (Parc Nationale permit DIR-I-2020-280 and Nagoya number ABSCH-IRCC-FR-254969-1). Beetles were kept alive in 50 ml Falcon tubes, with five per tube, water-soaked facial tissues for moisture, and punctured lids for aeration. After returning to our lab in Germany, we decapitated the beetles and placed them in wire-mesh cages (15 × 5 cm, 1.25 mm mesh and 0.4 mm wire). We added two carcasses per cage to ensure that each cage/carcass duo would be positive for both *P. mayeri* and *Acrostichus* (Figure 2).

For the additions of *P. pacificus* dauers, we made dauer cultures in lab following a modified version of liquid cultures described in Werner et al., 2017 (25% more eggs were added, and 25% less OP50 *Escherichia coli*) and using a fluorescently tagged derivative of the *P. pacificus* wildtype PS312 with 100% transmission rate, RS3832 (*tuEx333; daf-1p*:TurboRFP), to more easily differentiate our added worms from wild worms. Amphid neurons and other neurons are expressed in dauers, J2-J4, and adults. Images of a fluorescent *P. pacificus* dauer and an adult can be seen in Supplementary Figure S1, alongside a *P. mayeri* dauer and an adult with no amphid fluorescence. We used the laboratory ‘wild type’ strain PS312 rather than a newly isolated *P. pacificus* strain from La Réunion because obtaining a genomically integrated fluorescence, and thus a 100% transmission rate, has proved arduous (this reliable line took years). Also, PS312 is one of a few strains that is 100% Eu, an important asset for the experiment performed. The Sommer Lab can provide RS3832 upon request. Dauers were isolated after 10 days in liquid culture, using 1% sodium dodecyl sulfate and then washed with M9 (Cassada and Russell, 1975). To transfer dauers to the carcasses, a 10 μl water droplet was pipetted onto the beetle, and a dauer was transferred to the droplet *via* a worm pick. Confirmation of a successful transfer was verified by brushing the pick onto a nematode growth medium (NGM) agar plate afterword to confirm absence of a worm. We had two cage types: ones with one *P. pacificus* dauer added (Ppa1) and ones with 10 *P. pacificus* dauers added (Ppa10) (Figure 2), in order to cover two hypothesized initial population sizes on the beetles. To avoid ambiguity, throughout the text, “Ppa” refers to a cage type (i.e., how many *P. pacificus* dauers were added in the experiment: Ppa1 = one *P. pacificus* dauer added) while “*P. pacificus*” refers to the species in all other contexts.

Carcasses sat in the center compartments within the stainless-steel cylindrical cage of wire-mesh that allows for free movement of the worms. The cages were 15 cm long with a 5 cm diameter and made of 1.25 mm mesh and 0.4 mm wire. The compartment dividers were made of 0.5 mm mesh and 0.25 mm wire and were placed 5 cm apart within the column. Each cage was divided into three compartments: the center one with the carcasses and soil, and two outer ones with soil. All three compartments were filled with autoclaved gardening soil, so that the carcasses were completely immersed in soil (Einheitserde, Classic Profisubstrat) and kept at 17°C. Cages were watered to mimic the natural environment of the beetles.

We had three replicates for time points t = 1 day, 4 days, 1 week, 2 weeks, 4 weeks, and 6 weeks, and two replicates for t = 8 weeks. Each replicate (two carcasses together in the same cage) at each time point was exclusively used for that single time point; duos of carcasses were only screened once (i.e., the carcasses used at t = 6 weeks are different than the ones used at t = 4 weeks). Though, after initial phenotyping, carcasses were observed longer to ensure that if any species had not initially been detected, it was due to its slower emergence and not its absence in total on the carcasses. Thus, every replicate shown here was positive for all three species. Due to previous studies (Renahan and Sommer, 2021), our intended longest time point was t = 6 weeks (after which the populations dwindle), but since not all surplus cages were used as replacements, we then processed two replicates for each cage type (Ppa1 and Ppa10) at t = 8 weeks. While n = three replicates is seemingly low for an ecological study, *G. bupthalma* is a restricted endangered species and we are limited in the specimen we can take. Yet, our replicates had reproducible trends even with the expected variation among them. For example, in Ppa1 at t = 4 week, one replicate had double the number of *Acrostichus* dauers of another replicate (44 and 22, respectively), but the trend, that overall t = 4 weeks had more *Acrostichus* dauers than at t = 2 weeks, remained. Thus, we are able to maintain reproducibility with limited replicates while ensuring the wild beetle population remains intact.

### Nematode isolation, identification, and phenotyping

At the predetermined time points, three cages for each experiment type were disassembled (two cages for t = 8 weeks). The two beetle carcasses were transferred to NGM plates, where worms were screened, while worms from the soil were isolated using Baermann funnels (Viglierchio and Schmitt, 1983). All soil was funneled. Worms of all stages (except eggs) were screened for species, stage, and mouth form on a Zeiss Axioskop. Recovered dauer larvae were allowed to exit on food for proper species identification.

A subset of worms was sequenced for species authentication: worms were lysed and then a 1 kb region of the small subunit (SSU) rRNA gene was amplified in a polymerase chain reaction (PCR) using primers SSU18A (5′AAA​GAT​TAA​GCC​ATG​CAT​G-3′) and SSU26R (5′CAT​TCT​TGG​CAA​ATG​CTT​TCG-3′) (Blaxter et al., 1998). Using sequencing primer SSU9R (5′-AGC​TGG​AAT​TAC​CGC​GGC​TG-3′), Sanger sequencing was conducted by GENEWIZ in Leipzig. NCBI reference database was used for taxonomic identification. As a consequence of inadequate data, we have been unable to firmly conclude which *Acrostichus* species is present. Throughout the manuscript, we refer to all potentially present *Acrostichus* species collectively as “*Acrostichus.*”

### Light microscopy, statistics, and data visualization

For microscopic imaging, all specimens were mounted on slides with agarose pads containing sodium azide as a sedative. Worms were photographed using a Zeiss Axio Imager. Z1 light microscope with a Zeiss Plan-Apochromat 100 × 1.4 DIC objective. All image stacks were taken with a Zeiss Axiocam 506 mono CCD camera. Fluorescent images were obtained using a Lumencor SOLA SE II 365 with an LED light source. Statistical analyses were performed in the base and stats packages of R (ver. 4.1.1, 64-bit platform) (R Core Team, 2022). All line graphs and bar plots were generated with ggplot2 (ver. 3.3.6) (Wickham, 2016). Dots in the line graphs represent the mean average value across all replicates for a given time point and species. We calculated the standard error of each mean (SEM) and visualized the ±1 SEM ranges for each dot via translucent ribbons surrounding the lines. All final figures were created in Affinity Designer (ver. 1.10.5). Affinity Photo (ver. 1.10.5) was used to adjust microscopic images for lightness and contrast, and to colorize structures of interest (i.e., teeth of the worms). In an effort to make our plots accessible to people with color-vision deficiencies (CVDs) and color blindness, we exclusively used the scientifically-derived colormaps viridis and vik, which are provided in the viridis (ver. 0.6.2) and scico (1.3.0) packages, respectively (Garnier et al., 2021; Pedersen & Crameri, 2021).

## Results

### Successions of three species over 2 months on decaying host carcass

**FIGURE 3:**
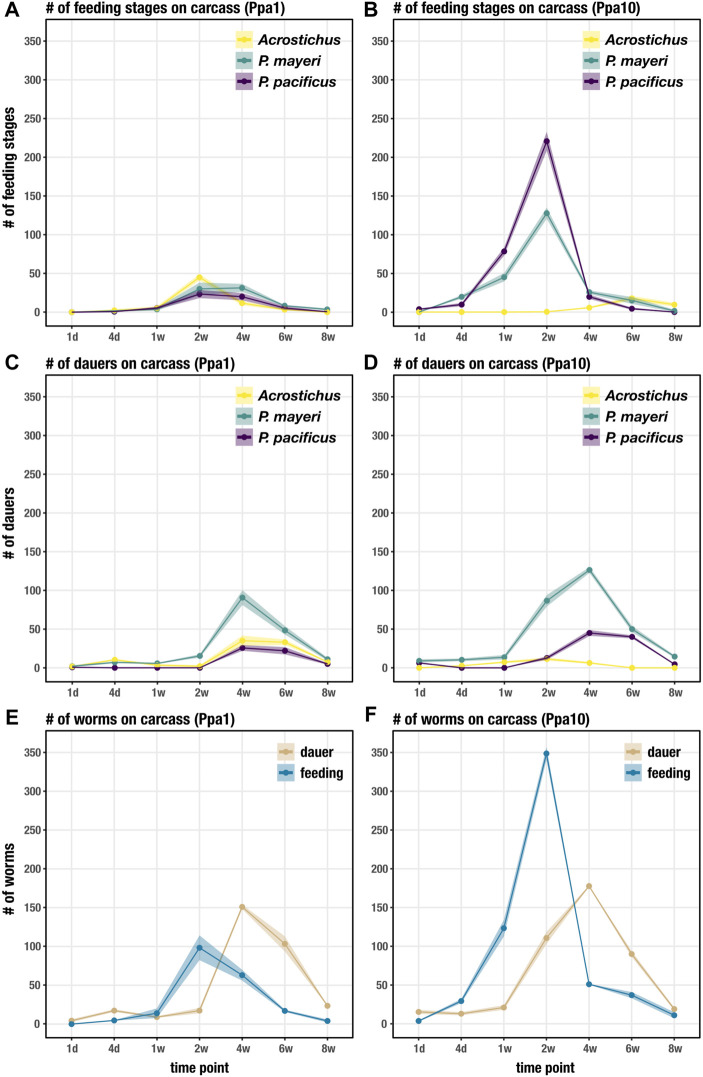
Succession of developmental stages on the beetle carcass over 8 weeks. **(A)** Succession of feeding stages of each species on Ppa1 carcasses over 8 weeks. **(B)** Succession of feeding stages of each species on Ppa10 carcasses over 8 weeks. **(C)** Succession of dauers of each species on Ppa1 carcasses over 8 weeks. **(D)** Succession of dauers of each species on Ppa10 carcasses over 8 weeks. **(E)** Succession of total feeding stages and dauers (the three species combined) on Ppa1 carcasses over 8 weeks. **(F)** Succession of total feeding stages and dauers (the three species combined) on Ppa10 carcasses over 8 weeks. N = 3 cages per time point (except t = 8 weeks, where n = 2 cages), with the average numbers shown. Ribbons indicate standard error of the mean.

We followed the dynamics of diplogastrids *P. pacificus*, *P. mayeri*, and *Acrostichus* over 2 months post-beetle death. Previous studies from our lab concluded that solitary *P. pacificus* on host *O. borbonicus* displayed a biphasic “boom and bust” pattern and were highly Eu (Renahan et al., 2021). In contrast, we found that *P. mayeri* when co-habiting with *Arcostichus*, 1) exited dauer between one and 2 weeks, 2) constantly maintained both feeding and dauer groups on the carcass, 3) had dauers continuously dispersing, and 4) were highly Eu only when crowded with *Acrostichus* (Renahan and Sommer, 2021). Here we show that when all three species are occupying the same putrefying insect, the dynamics change. This is primarily evident in Ppa10 environments, in which *P. mayeri* is quicker to exit dauer and start reproducing earlier (Figure 3B). In Ppa1, all three species have feeding stages by t = 2 weeks (Figure 3A), though by day 4 in Ppa10, both *Pristionchus* species have already exited dauer. The two *Pristionchus* species in Ppa1 maintain their highest feeding populations between weeks 2 and 4, while in Ppa10 both of their peak feeding populations occur at t = 2 weeks, after which their numbers decrease. Interestingly, *Acrostichus* has the highest peak of feeding stages in Ppa1, occurring at t = 2 weeks, while in Ppa10 *Acrostichus* does not start populating until t = 4 weeks, and never reaches the peaks that the two *Pristionchus* species do. In both experiments, feeding populations dwindle by t = 6 weeks, and have nearly ceased by t = 8 weeks. Thus, the artificial addition of *P. pacificus* affected both natural nematodes of the *Gymnogaster* ecosystem, with *P. mayeri’s* hastier dauer exit and more rapid development representing the most drastic change.

The dauer populations on the carcass of both *P. mayeri* and *Acrostichus* in Ppa1 during the first 2 weeks most likely consist of the dauers initially on the live beetle, with new dauers emerging after t = 2 weeks (Figure 3C). The added single *P. pacificus* dauers in Ppa1 exit dauer within a day, and no new dauers are seen until t = 4 weeks. Also on Ppa1, the three species have peak dauer populations around t = 4 weeks, and by t = 8 weeks very few dauers remain on the carcass. On Ppa10, *P. mayeri* dauers have already emerged from the carcass at t = 1 day (Figure 3D), faster than seen on Ppa1. In contrast to Ppa1, not all 10 *P. pacificus* dauers exit within a day on Ppa10. New *P. pacificus* dauers appear at t = 2 weeks on Ppa10. *Acrostichus* dauers do not emerge from the carcass until t = 4 days, and do not appear to have large populations of dauers at any time. After t = 4 weeks, the dauer abundance of all three species decreases. In both Ppa1 and Ppa10, *P. mayeri* has greater dauer numbers than the other two species. Notably, ten *P. pacificus* dauers triggered earlier emergence of *P. mayeri* dauers and daunted *Acrostichus* into hiding in dauer, behaviors we had not observed when *P. mayeri* and *Acrostichus* cohabited without *P. pacificus*.

Looking at the total worm numbers on the carcass, with the abundances of the three species combined for dauers and feeding stages, we see that there are fewer feeding worms overall on Ppa1 (Figure 3E) than on Ppa10 (Figure 3F). In both experiments, feeding stages reach their peaks at t = 2 weeks, though the abundance is over three times greater in Ppa10 (∼350 compared to ∼100). Although, at t = 4 weeks, the feeding population on Ppa1 is similar to its peak at t = 2 weeks (suggesting maintenance of a constant feeding stage population), while in Ppa10 the population dives by t = 4 weeks. The increase in dauers begins earlier in Ppa10, with the proliferation starting from t = 1 week, while in Ppa1 it starts after t = 2 weeks. Yet, in both Ppa1 and Ppa10, the dauer peaks still occur at the same time, t = 4 weeks, and both occur after the peak of feeding stages. Overall, there are more worms on Ppa10 than on Ppa1, most notably the feeding stage surge at t = 2 weeks. While the overall dauer numbers on the carcass are similar between Ppa1 and Ppa 10, there are more dauers dispersing earlier in Ppa10. Note that the exact number of worms on the carcass cannot by directly compared to the data obtained in the previous experiment (Renahan and Sommer, 2021), as these cages were established from single *Gymnogaster* beetles. Yet, the overall nematode succession trend seen here differs from the previous experiment: here, the high peak of feeding stages precedes the peak of dauers, while previously the two populations grew and diminished synchronously.

### Dauers disperse in surrounding soil

**FIGURE 4:**
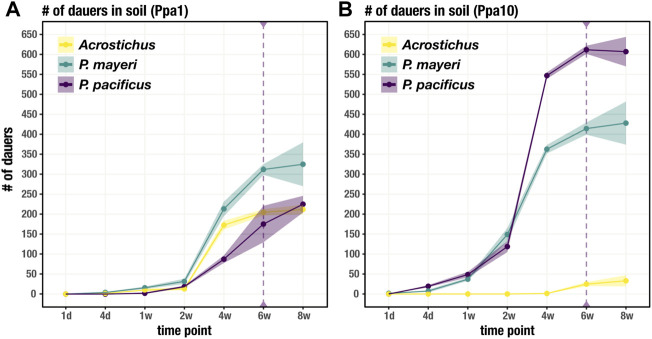
Dauer dispersal from the carcass to soil. Soil includes the soil immediately surrounding the carcass (in the carcass compartment) and the soil in the rest of the cage. Number of dauers found outside of the carcass for each species over 8 weeks on Ppa1 **(A)** and Ppa10 **(B)**. Dotted purple line at t = 6 weeks defines when *P. pacificus* was first found in soil outside of the carcass compartment, not just the carcass soil in the carcass compartment. N = 3 cages per time point (except t = 8 weeks, where n = 2 cages), with the average numbers shown. Ribbons indicate standard error of the mean.

While the feeding stages restricted themselves to the carcass, dauers dispersed into the surrounding soil. We tracked the movement of dauers for each species over the same time period as worm succession on the carcass. Unsurprisingly, it took time for dauers to appear in the surrounding soil, with numbers steadily increasing after t = 1 week in Ppa1 (Figure 4A). The three species dispersed in similar fashion in Ppa1, with *P. mayeri* dominating in number of dauers. Over time, the number of dauers in the surrounding soil increases. In Ppa10, dauers begin dispersing a bit earlier than in Ppa1, starting around t = 4 days (Figure 4B). In Ppa10, while the two *Pristionchus* species leave the carcass, *Acrostichus* is not seen in the surrounding soil until t = 6 weeks, consistent with its lack of high populations on the carcass. Overall, the numbers of dispersing dauers reflect the abundances of the species on the carcass. Interestingly, the two carcass-derived species, *P. mayeri* and *Acrostichus*, were found outside the carcass compartment as early as t = 2 weeks (only in Ppa1 for *Acrostichus*). In contrast, the laboratory species *P. pacificus* was not found in the soil of the carcass compartment until t = 6 weeks. Thus, when they had dauers, the carcass-derived worms dispersed further, earlier.

### Successions of *Pristionchus* mouth forms over 2 months

**FIGURE 5:**
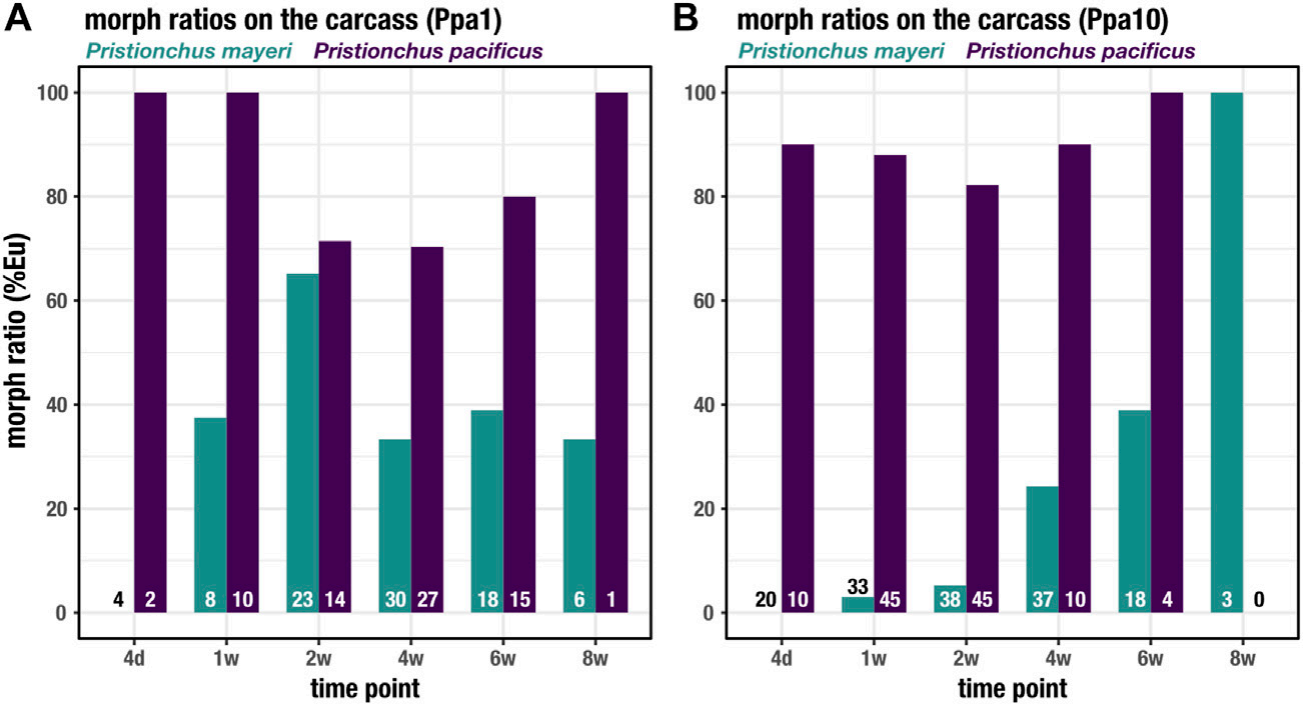
Mouth forms of *Pristionchus* species on the carcass. The percent Eu for *P. pacificus* and *P. mayeri* over 8 weeks on Ppa1 **(A)** and Ppa10 **(B).** N is indicated at the bottom of the bars for each species and time point.

Both *Pristionchus* species displayed the mouth-form dimorphism, unlike *Acrostichus*, and were tracked over the 8 weeks on the beetle carcass. In both Ppa1 (Figure 5A) and Ppa10 (Figure 5B), *P. pacificus* was highly Eu (70–100%), though under laboratory conditions this strain is typically 95–100% Eu (Werner et al., 2017; Sieriebriennikov et al., 2020). The lowest rate of Eu occurred on Ppa1 between t = 2 weeks and t = 6 weeks, corresponding to the highest populations of feeding stages (Figure 3E). While on Ppa10, the *P. pacificus* Eu rate remained above 80% over the 2 months, though it was only 100% after the plummet from the population proliferation. In Ppa1, *P. mayeri* increases from 0% Eu at t = 4 days up to 65% at t = 2 weeks, after which it decreases but remains between 30 and 40% from t = 4 weeks to t = 8 weeks. The peak Eu incidence of *P. mayeri* at t = 2 weeks coincides with the peak of *Acrostichus* feeding stages (Figure 3A). On Ppa10, *P. mayeri* steadily increased its Eu ratio, starting at 0% at t = 4 days and reaching 100% by t = 8 weeks. While these high Eu occurrences may be reflective of the presence of *Acrostichus* after t = 4 weeks (Figure 3B), the extremely high frequency at t = 8 weeks might be influenced by the small sample size. Overall, *P. pacificus* Eu percentages decreased when populations were most dense, while *P. mayeri* had its highest Eu ratios when *Acrostichus* abundance was at its highest, and lower rates when Ppa10 was crowded with *Pristionchus* species. Markedly, in previous studies in which *P. pacificus* was absent, *P. mayeri* only developed as predators in populations dense with *Acrostichus*.

## Discussion

We investigated the employment of plastic traits in three diplogastrid species cohabitating in a short-lived natural ecosystem. Utilizing a malleable system, beetle *G. bupthalma* and its symbionts *P. mayeri* and *Acrostichus*, we added a third competitor, model nematode *P. pacificus*, to explore how the dauer and mouth-form polyphenisms are strategically used over the course of 2 months post-beetle death. We experimentally manipulated the system by adding either one *P. pacificus* dauer (Ppa1) or ten *P. pacificus* dauers (Ppa10), in an effort to mimic two potential initial populations on a beetle. We observed the dynamics on the carcass, phenotyping developmental stages and tracking the formation and dispersal of dauers from the carcass to surrounding soil. The succession of mouth forms revealed that only the two *Pristionchus* species displayed the dimorphism, as *Acrostichus* remained exclusively non-predatory. Overall, we conclude that the three species each uniquely responds to competition.

*Pristionchus pacificus* in the face of competitors behaves differently than when its devouring an insect carcass in isolation; alone, *P. pacificus* displays a biphasic “boom and bust” pattern (Renahan et al., 2021), but here, *P. pacificus* consistently maintained populations of both feeding stages and dauers (Figure 3). The species heavily proliferated in Ppa10 compared to its reproductive endeavors in Ppa1, which likely reflects its starting population sizes. The population sizes of *P. pacificus* overall were surprisingly low, especially in the first few time points, as its brood size is well-established in lab (120-180 viable eggs in PS312; ∼120 in RS3832, our tagged PS312 line) and we expected higher numbers (Sommer et al., 1996; Pires-daSilva, 2013). Though, the *P. pacificus* PS312 strain used in this experiment has been domesticated for over 2 decades and may not thrive well in a wild environment. Perhaps it has acclimatized to an abundance of food and nutrients available at its cuticular tips, and has lost its ability to cope with limited bacteria. Potentially however, the abundance of bacteria might not be the issue, but the bacterial type, which may explain why *P. pacificus* is not regularly found on *G. bupthalma.* In addition, some beetle hosts produce pheromones antagonistic to nematode presence, inducing embryonic arrest and inhibiting dauer exit, that may affect population sizes (Cinkornpumin et al., 2014; Renahan and Hong, 2017). *Pristionchus pacificus* dauers were not as prone to actively disperse as beetle-derived dauers (until t = 6 weeks, they were restricted to the beetle compartment) (Figure 4), suggesting another effect of domestication. Indeed, bacteria influence dauer characteristics, and a domesticated strain may be insensitive to those stimuli (Samuel et al., 2016; Bubrig et al., 2020). Unexpectedly, *P. pacificus* did not display exclusively high rates of Eu (Figure 5), in contrast to its morph ratios in lab, >95% (Werner et al., 2017; Sieriebriennikov et al., 2020). While Eu *P. pacificus* can relatively easily predate on *P. mayeri,* they often struggle ripping apart the tough cuticle of *Acrostichus*. Given the unusual prevalence of St mouths on the carcass, we speculate that *P. pacificus* is potentially employing bet hedging as a strategy in competitive environments with partially unpredictable succession. Together, this study revealed several new features and behaviors of *P. pacificus* that differ from both animals reared in the laboratory on *E. coli* monoxenic cultures and ones examined in their natural environment.

*Pristionchus mayeri* responded to the two different initial populations of *P. pacificus* distinctively. In Ppa1, *P. mayeri* produced small populations, though invested in Eu worms (Figure 3A, Figure 5A). In contrast, *P. mayeri* in Ppa10 had larger populations, reflecting its speedy development and more St adults (Figure 3B, Figure 5B). The brood size for these carcass-derived *P. mayeri* on NGM agar plates is ∼100 viable eggs. In Ppa10, *P. mayeri* did not begin developing many Eu worms until the population decreased at t = 6 weeks. Indeed, previous studies in *P. pacificus* concluded that development to reproductive maturity is quicker for St worms than Eu worms, implicating a fitness trade-off between dietary range and developmental speed (Serobyan et al., 2013). Thus, in dense *P. pacificus* competitor settings, *P. mayeri* favored rapid reproduction over predatory development. High Eu rates in *P. mayeri* also coincide with presence of *Acrostichus.* While this is consistent with previous studies detailing *P. mayeri* competing only with *Acrostichus* (Renahan and Sommer, 2021), the primary mouth-form strategy of *P. mayeri* when faced with two competitors is in response to its congeneric.

*Acrostichus* as well displayed two disparate strategies in response to the two populations of *P. pacificus*. While on NGM agar plates *Acrostichus* produces comparative brood sizes (100-150) to the two *Pristionchus* species, on the carcasses the populations were rather low. In Ppa1, *Acrostichus* remained competitive, increasing its population slowly and within the ranges of the two *Pristionchus* species. It also maintained a population of dauers on the carcass while continuously dispersing. Yet, in Ppa10, *Acrostichus* did not participate in combat. Hiding in dauer as the two *Pristionchus* species dueled, *Acrostichus* did not emerge to feed until t = 4 weeks, when the *Pristionchus* populations dwindled. Evading engagement may have had its inconspicuous benefits, but upon dauer exit, by 4 weeks food limitations restricted population growth. Perhaps *Pristionchus* population growth reflects their acquired cellulases, enabling manipulation of the microbial community (Mayer et al., 2011; Han et al., 2022). Intriguingly, we never observed the Eu morph in *Acrostichus*. This could be due to a lack of the dimorphism in the species, which varies in the genus (Giblin and Kaya, 1984; Ahlawat and Tahseen, 2016), or an inability to induce the Eu morph in these settings. The delayed dauer exit in Ppa10 and the lack of Eu worms overall can potentially be explained by nematode-derived modular metabolites (NDMMs), small molecules that facilitate communication among worms, including inducing plastic responses and regulating behaviors (Butcher et al., 2007; Bento et al., 2010; Bose et al., 2012; Falcke et al., 2018; Dong et al., 2020). This includes dauer entry, exit, and dispersal, and induction of the Eu form; these triggers can happen both inter- and intraspecifically, complicating our ability to pinpoint responsible players of plastic responses (Mayer and Sommer, 2011; Bose et al., 2014; Mayer et al., 2015; Werner et al., 2018).

Implications of these results can be interrupted as convoluted or trifling, the latter a caveat due to the semi-artificial aspect of the experiment inherent in its set-up. While this limitation is acknowledged, these findings may be predictive of the likely future natural encounters among *P. pacificus*, *P, mayeri*, and *Acrostichus*. The *G. bupthalma* habitat on La Réunion Island is mere meters away from the territory of *Adoretus* beetles, a known *P. pacificus* host (Morgan et al., 2012; Gomy et al., 2017). In recent years, we have found the occasional *P. pacificus* on *G. bupthalma* and *P. mayeri* on *Adoretus*, potentially due to slightly overlapping geographical ranges of the two hosts. In addition, we have identified a second locality on the island crawling with *G. bupthalma*; we will soon determine the species richness of nematodes on these beetles. Thus, this seemingly factitious ecosystem is consistent with observed trends. And now that we have established how these wild strains behave in a natural setting, we can next determine the life history traits of undomesticated worms in a laboratory context. Furthermore, regardless of the reality of *P. pacificus* as a natural competitor, we show that *P. mayeri* develops distinct phenotypes in response to different competitors, reflecting the evolutionary and ecological importance of plasticity.

Overall, in a competitive environment with limited food, nematodes employ plastic phenotypes to ensure success. Equipped with dauer and mouth form, diplogastrids maintain options in which strategies to utilize, and our experimental manipulation of a natural ecosystem revealed species-specific responses to competition. Added competitor *P. pacificus* optioned for bet hedging as it developed both mouth forms, while natural rivals *P. mayeri* and *Acrostichus* proliferated quickly and ensconced in dauer, respectively. Determining a winner on the carcass is a challenging task, as what constitutes championship? Is it the species with the largest reproducing population, or the one that has the most dispersing dauers, ensuring encounters with prospective new hosts? Bestowing a victor is not trivial due to the heightening complexity of ecosystem functioning, including the many factors influencing dauer characteristics and mouth form, from bacteria to NDMMs. In culmination, the competitive worm indeed mounts through several plastic spires of form as it strives to thrive in a multidimensional beetle ecosystem.

## Data Availability

The original contributions presented in the study are included in the article/Supplementary Material, further inquiries can be directed to the corresponding author.
