# Supplementary figures and images for: Multidimensional competition of nematodes affects plastic traits in a beetle ecosystem
Source: Front Cell Dev Biol. 2022 Aug 24;10:985831. doi: 10.3389/fcell.2022.985831 (PMC9449363; doi:10.3389/fcell.2022.985831)

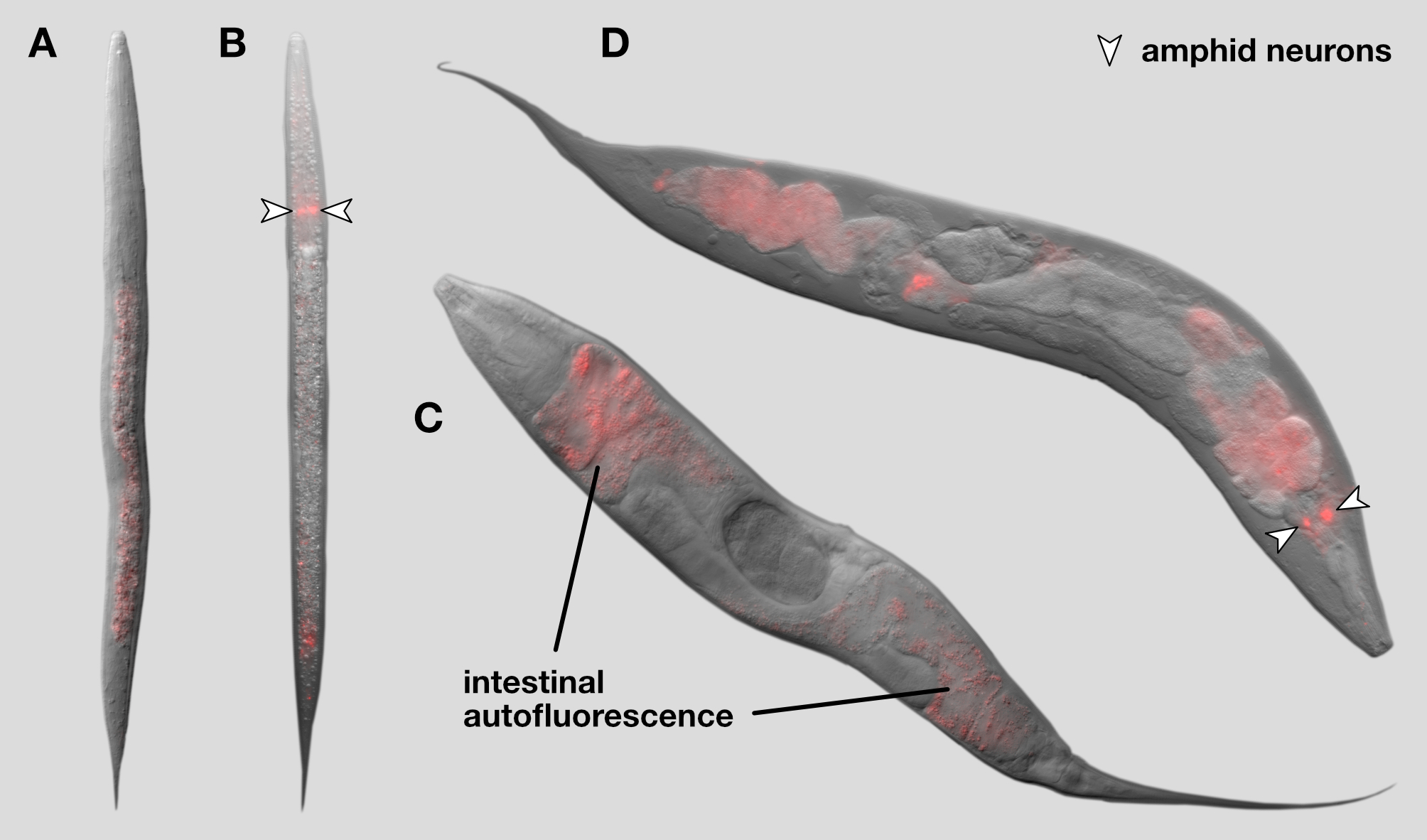

Supplement: Supplementary file 4 [file Image1.JPEG]
